# Memory B cells and long-lived plasma cells in AMR

**DOI:** 10.1080/0886022X.2022.2128374

**Published:** 2022-10-03

**Authors:** Wenlong Yue, Jia Liu, Xiaohu Li, Luman Wang, Jinfeng Li

**Affiliations:** a Kidney Transplantation Unit, The First Affiliated Hospital of Zhengzhou University, Zhengzhou, China; b Dietetics Teaching and Research Section, Henan Medical College, Xinzheng, People’s Republic of China; c Department of Immunology, School of Basic Medical Sciences, and Institutes of Biomedical Sciences, Fudan University, Shanghai, China

**Keywords:** Antibody-mediated rejection, memory B cells, long-lived plasma cells

## Abstract

Antibody-mediated rejection (AMR) has a strongly negative impact on long-term renal allograft survival. Currently, no recognized effective treatments are available, especially for chronic antibody-mediated rejection (CAMR). Donor-specific antibodies (DSAs) secreted by long-lived plasma cells and memory B cells are acknowledged as biomarkers of AMR. Nevertheless, it may be too late for the DSA routine examination production since DSAs may have binded to graft vascular endothelial cells through complement-dependent or complement-independent pathways. Therefore, methods to effectively monitor memory B cells and long-lived plasma cells and subsequently prevent DSA production are key to reducing the adverse effects of AMR. Therefore, this review mainly summarizes the production pathways of memory B cells and long-lived plasma cells and provides suggestions for the prevention of AMR after transplantation.

## Introduction

AMR is mainly mediated by DSAs, which are the main cause of allograft dysfunction. There are currently no recognized effective treatments for AMR [1,2]. Activating naive B cells interact with CD4^+^ T cells and differentiate into memory B cells and long-lived plasma cells, which produce antibodies through germinal center (GC) -dependent or GC-independent pathways upon antigen stimulation in the human body. In terms of transplantation, antibodies secreted by long-lived plasma cells induce inflammatory damage to graft vascular endothelial cells through complement-dependent or complement-independent pathways, and this damage is considered to be the main contributor to the pathogenesis of AMR [3,4]. Humoral rejection responds poorly to existing immunosuppressive drugs. Plasma exchange and immunoglobulin injection are commonly used clinical methods to remove DSAs, but they cannot effectively remove memory B cells or long-lived plasma cells, which may be an important reason why CAMR is difficult to cure [5]. In this paper, we first discuss the production pathways of memory B cells and long-lived plasma cells as well as the heterogeneity of memory B cells and then propose potential preventive targets for AMR from the perspective of T-B-cell interactions with the aim of providing insight into finding ways to prevent and treat AMR.

## Generation of memory B cells and long-lived plasma cells

B-cell precursors differentiate in the bone marrow microenvironment, proliferate, express pre-B-cell receptors (pre-BCRs), reproliferate, express antigen receptors, and undergo positive and negative selection, ultimately leading to the generation of mature B cells [6–8]. When B-cell precursors develop into mature B cells, the resulting B cells present with different morphological and functional characteristics. Most maturing B cells undergo apoptosis during positive or negative selection. Few B cells undergoing functional immunoglobulin gene rearrangement enter peripheral lymphoid organs, and ultimately develop into juvenile B cells [9–12].

**Figure 1.**
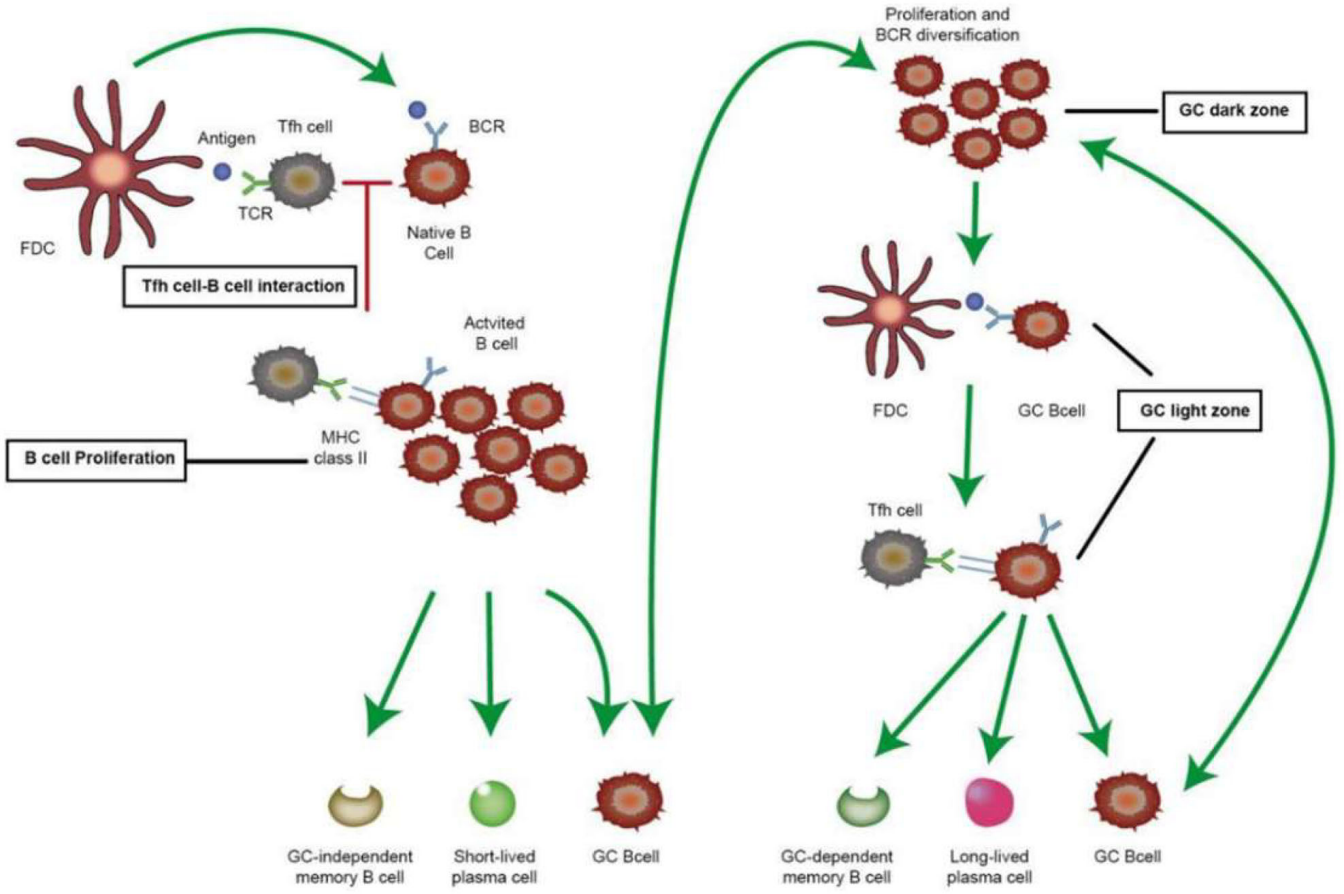
Production pathways of memory B cell and long-lived plasma cells. Immature B cells are activated by antigens and migrate to B-cell follicles in secondary lymphoid organs. The same antigen is processed by antigen-presenting cells (APCs) and presented to special CD4^+^ T cells (follicular helper T (T_FH_) cells). T_FH_ cells migrate to the T-cell-B-cell boundary to establish stable T-cell-B-cell interactions. With the help of T_FH_ cells, activated B cells undergo one of three fates. Some B cells become short-lived plasma cells and rapidly produce specific antibodies against pathogens. Other B cells develop into memory B cells (GC-independent memory B cells). Activated B cells that do not differentiate into plasma cells return to the B-cell follicle state and undergo rapid proliferation to form unique structures with T_FH_ cells, namely, GCs. GCs are divided into two parts: a light zone and a dark zone. In the dark zone of GCs, antigen-specific B cells proliferate and diversify with BCRs undergoing a high rate of mutation in vitro. B cells then leave the dark zone and enter the light zone. Follicular dendritic cells (FDCs) continuously stimulate B cells with antigens and interact with T_FH_ cells that migrate to the GC, generating three pathways: the long-lived plasma cell production pathway, the GC-dependent memory B-cell production pathway and the dark zone pathway in the GC that consumes and produces GC B cells. TCR: T-cell receptor; BCR: B-cell receptor; FDC: follicular dendritic cell.

It has been traditionally believed that memory B cells are produced mainly through T-cell-dependent immune processes, usually in response to the presence of protein antigens [13]. These mechanisms have been described in detail [14–17]. Specifically, in this process, immature B cells activated by antigen-presenting cells (such as follicular dendritic cells (FDCs)) and special CD4^+^ T cells (such as follicular helper T (T_FH_) cells) can establish stable interactions with T-B cells [18,19], which promotes the proliferation and differentiation of immature B cells into one of three main cell types: GC-B cells, short-lived plasma cells and GC-independent memory B cells. Among these types, short-lived plasma cells can rapidly produce specific antibodies against pathogens. The relevant literature has indicated that these short-lived plasma cells accumulate in the red pulp of the spleen and the medullary cords of lymph nodes, generally only during the process of infection [20]. The newly generated GC B cells form the GC and undergo proliferation and B-cell receptor (BCR) mutation in the dark zone, after which they enter the light zone, where they are stimulated by antigens presented by FDCs and interact with T_FH_ cells that have migrated to the GC, which leads to one of three outcomes: differentiation into GC-dependent memory B cells, differentiation into long-lived plasma cells or recirculation into the dark zone of the GC (Figure 1) [21–23]. In the context of transplantation, when HLA antigens stimulate the human body, activated naive B cells generate memory B cells and long-lived plasma cells through the abovementioned GC reaction, forming the immune memory function of the body.

The fate of GC B cells that differentiate into plasma cells is worthy of further study. Plasma cells are differentiated from activated B cells. Studies have shown that bcl-6^low^CD69^high^GC B cells expressing the transcription factor IRF4 are more likely to differentiate into plasma cells than those without IRF4 expression [24]. These B cells in the GC, which express high levels of ICAM1 and SLAM, are regulated by CD40 signaling that is mediated by T cells, which emphasizes the importance of the interaction between T_FH_ cells and B cells [25,26].

Recent studies have shown that memory B cells can be produced in a T-cell-independent manner in the abdominal cavity, where B cells are often abundant, but the frequency of T-cell-independent memory B-cell generation in secondary lymphoid organs, such as the spleen, is low; cells that are generated in this manner are known as B1 cells [27,28]. B1 cells can be activated only by polysaccharide antigens. Most memory B cells express IgM and exhibit the characteristics of many immature B1 cells, mainly because of the relative infrequency of homotypic conversion or somatic hypermutation [29,30]. Studies have also shown that IgM^+^ T-cell-independent memory B cells are directly derived from multipotential precursor cells in the early immune response [31] and generate GC-dependent memory B cells in the late immune response through homotypic conversion. Therefore, memory B cells can be produced in a T-cell-dependent or T-cell-independent manner [32–34]. However, differences between T-cell-independent memory B cells and naive B cells in terms of their antigen response, including whether the response of naive B cells is as fast and strong as that of T-cell-dependent memory B cells, are unclear, and need further study. Such cells may be related to the production of DSAs and AMR, and further research may help to understand the role of T-cell-independent memory B cells in AMR.

## Heterogeneity of memory B cells

There are different types of memory B cells, such as IgG^+^, IgM^+^, IgA^+^, and IgE^+^ memory B cells. Moreover, each memory B-cell type has a unique function [35,36]. Here, we mainly discuss IgG^+^ and IgM^+^ memory B cells. As mentioned above, in the early stage of antigen exposure, memory B cells are generated independent of the GC, and these early cells are most likely to be IgM^+^ memory B cells. GC B cells show a high degree of somatic hypermutation and type conversion and are more likely to differentiate into IgG^+^ memory B cells than into IgM^+^ memory B cells [37–39]. The functional differences between IgM^+^ and IgG^+^ memory B cells are partially determined by the BCRs that they express; for example, BCRs on IgG^+^ memory B cells contain 28 amino acid residues in their cytoplasmic tail, while BCRs on IgM^+^ B cells contain only 3 amino acid residues in their cytoplasmic tail [40–42]. Another potential explanation is that they express different transcription factors. For example, some biochemical studies have shown that DLG1 (also known as SAP97) can promote the proliferation of the B-cell junction protein growth factor receptor-binding protein 2 (Grb2) and membrane-associated guanylate kinase (MAGUK) family proteins that bind to the IgG cytoplasmic tail and can promote the proliferation of B cells [43]. The expression level of the transcription factor BACH2 in IgG^+^ memory B cells is lower than that in IgM^+^ memory B cells. BACH2 can inhibit plasma cell differentiation [41,44]. Therefore, in this study, during reinfection, compared with IgM^+^ memory B cells, IgG^+^ memory B cells showed a stronger antigen response and were more likely to differentiate into plasma cells, while IgM^+^ memory B cells showed higher proliferation rates and were more likely to enter the GC, similar to naive B cells [45]. Notably, the molecular markers on the surface of IgG^+^ and IgM^+^ memory B cells are also very different, which may explain the functional differences between these two types of memory B cells [46]. In memory B cells that interact with T cells, the expression of CD73, CD80 and programmed cell death ligand-2 (PDL-2) in IgG^+^ memory B cells was higher than that in IgM^+^ memory B cells [47–49]. Therefore, it could be concluded that IgG^+^ memory B cells are more likely to differentiate into plasma cells than IgM^+^ cells are, while IgM^+^ memory B cells are more likely to reenter the GC. These explanations show that the different phenotypes of memory B cells lead to different cell fates. When sensitized patients are transplanted, IgG + memory B cells interact with memory Tfh cells to rapidly generate antibody-secreting cells (ASCs) and produce DSAs, which promotes the occurrence of AMR, while IgM + memory B cells are more inclined to enter the GC. Therefore, differences in memory B-cell types may determine changes in DSA concentrations.

## Memory B cell immunology

The human immune system exerts an enhanced effect on re-encountered antigens. Memory B cells are preferentially activated over naive B cells and differentiate into plasma cells when re-exposed to allogeneic antigens, and activated memory B cells secrete new DSAs, eventually leading to antibody-mediated graft dysfunction. For example, in sensitized patients undergoing secondary transplantation, many new DSAs can be rapidly produced [3,50]. This outcome is mainly a result of the somatic hypermutation of memory B cells in the GC process, which enables them to bind more tightly to antigens [44,51,52]. Therefore, effective monitoring of memory B cells before graft rejection is beneficial for predicting the occurrence of AMR, which is a major challenge that we need to address.

Moreover, relevant studies have shown that memory B cells are produced much earlier than long-lived plasma cells; as a consequence, the memory B-cell-mediated DSA response cannot be effectively resolved [53]. As a result, panel reactive antibody (PRA)-negative patients are often presumed to be unsensitized patients. The patient’s degree of sensitization is based on detection of PRAs. Nevertheless, PRA-negative patients with a large number of memory B cells quickly produce DSAs when exogenous antigen reintroduction occurs. These patients may be clinically classified as unsensitized patients. Therefore, AMR can still occur after transplantation in some PRA-negative patients, perhaps because of the impact of memory B cells [54,55]. Quantifying memory B cells in peripheral blood and GCs and determining their functions may be important approaches to monitor AMR [56].

Currently, there are no widely utilized or approved methods to detect various memory B cells in the clinic [57]. However, Wehmeier et al. [58] summarized several methods for testing memory B cells: flow cytometry analysis of HLA tetramer-positive B cells, Luminex SAB analysis of the culture supernatant of B cell stimulated by polyclones, and ELISpot analysis of memory B cells producing DSAs. Together, these methods well summarize the experience of detecting memory B cells and have important clinical guiding significance in transplantation rejection. Additionally, Lucia et al. [59] found that in patients waiting for renal transplantation, the assessment of HLA-specific B cells with the ELISpot method could detect the number of peripheral blood memory B cells, which has predictive value for the risk of AMR after transplantation.

## Plasma cell immunology

Plasma cells are differentiated from activated B cells. As the final B cells to be produced, plasma cells can be classified into short-lived plasma cells and long-lived plasma cells from GC [60]. Long-lived plasma cells produce high-affinity, class-switched antibodies, while memory B cells have broader antigen specificity. Therefore, when antigens enter the human body, long-lived plasma cells produce neutralizing antibodies against antigens in the first phase of immune response. In the second phase of immune response, memory B cells play important roles by rapidly producing antibodies with high affinity against various pathogens [50,55]. In terms of surface marker expression, CD38 and CD138 generally colocalize on the plasma cell surface [61]. Garimalla et al. [62] reported ASCs, including plasma cells and long-lived plasma cells, in peripheral blood 7 days after tetanus vaccination. These ASCs lacked CD20 expression. CD20 is specifically expressed in the late stage of B-cell development, and the failure to detect CD20 may be an indication of successful B- cell conversion into plasma cells [63,64]. After clinical transplantation, mature GC B cells and memory B cells can be eliminated from patients with AMR with drugs targeting CD20 (such as rituximab), but this treatment cannot effectively reduce the concentration of DSAs, which is consistent with the absence of CD20 molecules on the surface of plasma cells.

Rituximab is a monoclonal antibody (mAb) that specifically targets the B-cell surface antigen CD20 and kills corresponding target cells. Cohen et al. [65] found that rituximab can clear more than 90% of CD20+ B cells in peripheral blood within 1–2 days and can also inhibit B cells in the spleen and lymph nodes. Although rituximab reduces the number of memory B cells, the long-lived plasma cells produced by B cells that are not depleted are stored in the bone marrow. They lack the CD20 marker, are not affected by rituximab, and can produce a steady stream of antibodies. Therefore, rituximab should not be used to reduce serum DSA concentrations in the event of AMR. In clinical application, rituximab is often used for preoperative induction, and the appropriate dose is selected according to patient body weight. Additionally, because rituximab kills other B cells with normal immune function, special attention should be paid to the side effects of rituximab, such as infection, gastrointestinal reactions, and cytopenias [66,67]. Therefore, the removal of long-lived plasma cells may be a focus of future research.

## Interaction between T cells and B cells

T_FH_ cells play an indispensable role in the T-cell-dependent memory B-cell response and GC response. T_FH_ cells express the transcription factor Bcl-6, which is an important marker for distinguishing CD4 ^+^ T cells [68,69]. Studies have shown that when an antigen repeatedly stimulates the human body, T_FH_ cells stimulate memory B cells to produce an enhanced effect, and the process by which memory B cells receive antigen signals and differentiate into plasma cells requires the participation of the cytokines IL-4 and IL-21 [70]. In addition, these two cytokines are known to be key regulators of B- cell differentiation, and T_FH_ cells are sources of these cytokines, emphasizing the importance of T_FH_ cells in memory B-cell activation. Furthermore, T_FH_ cells provide an important helper signal for B cells that bind the same antigen. After T_FH_ cells are activated by antigens, direct intercellular signals between ICOS, CD28, and PD-1 molecules on the surface of T_FH_ cells and CD80/86, CD40, PDL-1 and other molecules on the surface of B cells, as well as the LFA-1/ICAM-1 integrin pathway and cytokines IL-4 and IL-21, lead to the upregulation of CXCR5 and the downregulation of CCR7. These factors promote the role of T_FH_ cells at the T-cell-B-cell boundary, their entrance into B-cell follicles and ultimately their entrance into the GC to participate in another round of reactions [71,72]. It has been reported that blocking the interaction between these costimulatory molecules may block DSA production and possibly preempt AMR in transplant recipients [73,74]. More interestingly, the contact time between T cells and B cells during this interaction affects the fate of B cells. B cells that maintain a long contact time present more antigenic signals to T_FH_ cells, and these B cells are more likely to enter the GC. In contrast, B cells that maintain a short contact time differentiate into short-lived plasma cells or GC-independent memory B cells, as fully verified in mouse experiments [15,75]. T_FH_ cells that enter the GC can secrete chemokine 13 (CXCL13), thus inducing more CXCR5^+^ B cells to enter the GC to promote the GC response, thereby producing memory B cells and plasma cells with high affinity [76,77]. Considering these findings, it can be speculated that, in clinical practice, blocking the CXCL13-CXCR5 axis may be a potential method for preventing DSA production after transplantation. In summary, T_FH_ cells play important roles in guiding B-cell differentiation, maturation and antibody class conversion in GCs and maintaining the normal operation of GCs and play an important role in all aspects of memory B-cell generation [78]. The final differentiation of B cells into memory B cells is not induced by a single molecular signaling process but is the result of the synergistic effects of multiple signaling molecules secreted by T_FH_ cells. However, more exploration is needed to identify the molecules that play leading roles in this process.

## Treatment recommendations for reducing antibodies from the perspective of memory B cells and long-lived plasma cells

### Eliminating DSAs in AMR

In terms of transplantation, the main cause of AMR is mismatch between the donor and recipient human leukocyte antigen (HLA) molecules. HLA is an expression product of the major histocompatibility complex (MHC), the products of which can be divided into HLA class I, class II and class III molecules. Class III molecules mainly include complement, which is mainly involved in the inflammatory response. Among them, the class I molecule and class II molecule are often tested clinically. Class I molecules include HLA-A, HLA-B and HLA-C, while Class II molecules include HLA-DR, HLA-DQ and HLA-DP [79]. The occurrence of postoperative AMR is essentially the production of antibodies against these mismatched HLAs. Therefore, it is very important to detect the number of mismatching of different epitopes of HLA in donors and recipients before surgery.

In patients with AMR, the DSAs produced *in vivo* are mainly anti-HLA and anti-non-HLA [59,80]. In the clinic, plasma exchange/immunoadsorption, as well as the injection of immunoglobulin-neutralizing antibodies, is often used to reduce plasma antibody levels in an effort to prevent graft rejection. Complementary pathway inhibitors [81], such as eculizumab, can also be used to alleviate the inflammatory pathological effects of antibody FC binding to complement proteins [82,83]. These inhibitors can reduce the incidence of rejection, to some extent, but the immune system undergoes a series of GC reactions that produce long-lived plasma cells that can persist in bone marrow for decades, even a lifetime, after kidney transplantation [51,64]. Therefore, the abovementioned medical intervention is only a temporary solution. It is an effective method to monitor the activity of long-lived plasma cells and memory B cells before the production of DSAs; subsequently, we can inhibit the body’s immune system through multiple immunouppressive regiments.

### Blocking T-B-cell interactions

In kidney transplant recipients, DSAs are important factors that affect kidney function [84]. Through a series of processes in the GC, memory B cells and long-lived plasma cells are produced, and these cells are the main sources of DSAs [85,86]. Therefore, we can target T-cell-B-cell interactions with compounds such as belatacept, a known costimulatory blocker of CD28 receptors that can inhibit binding of the costimulatory molecule CD28 to its ligand, CD80/CD86, on T cells, thereby specifically blocking signal 2 of the T-cell cycle, resulting in a significant reduction in T-cell activation or proliferation and subsequent prevention of DSA production and AMR [87,88]. In addition, other costimulatory molecules, such as CD40-CD154, are considered potential therapeutic targets. The CD40-CD154 costimulatory signaling pathway can promote the proliferation of B cells, antibody production, antibody class conversion in the GC and the generation of memory B cells in the antigen-induced immune response dependent on T cells [89]. The blocking of this signaling pathway is generally achieved by two means: anti-CD154 agents and anti-CD40 agents. Animal studies have shown good results for anti-CD154 agents, such as toralizumab and ruplizumab, in inhibiting rejection and inducing immune tolerance. However, in clinical experiments of anti-CD154, Law et al. [90] found that patients showed arteriovenous thrombosis as a side effect, which may be related to the activation of CD154 on the platelet surface. Anti-CD40 antibodies can avoid such complications. Therefore, an increasing number of anti-CD40 drugs, such as Chi220, 2C10, and ASKP1240, have been used in animal experiments. In islet transplantation, Chi220 can prolong the survival time of the graft, but its long-term use increases cytomegalovirus (CMV) infection and also leads to a decrease in the number of B cells, thereby causing a further increase in the infection rate [91]. 2C10 is a human anti-CD40 antibody that does not deplete B cells and increases graft survival in islet transplantation [92]. In a kidney transplantation model, ASKP1240 not only prolonged the survival rate of transplanted kidneys, but also inhibited the production of DSAs. Additionally, it has shown good effects in human clinical experiments [93].

Many studies have shown that PD-1 plays an indispensable role in the response of the GC. PD-1 is a known coinhibitory molecule [94,95] that can inhibit the aggregation of T_FH_ cells in the GC. However, this molecule is highly expressed in T_FH_ cells in the GC, which would seem to counteract its function. This contradiction was previously resolved with the explanation that PD-1 can form a mutually antagonistic complex with ICOS to drive T_FH_ cells with high ICOS expression into the GC [96]. However, a recent excellent review showed that the PD-1 molecule also has a positive effect; that is, it can limit the interference of CXCR3 and thus contain T_FH_ cells into the GC [97]. It is important for us to know whether PD-1 inhibitors can overcome tumor treatment obstacles and be applied in patients experiencing transplant rejection. Studies have shown that the chemokine CCL22 is essential for the affinity maturation of GC B cells. A higher number of T_FH_ cells better facilitates B-cell expression of CCL22; that is, the level of chemokine CCL22 can control the frequency of contact between B cells and T_FH_ cells to some extent, and for B cells with strong antigen affinity, CCL22 may be highly expressed, triggering more T_FH_ cell helper signals [98,99]. This mechanism may be applied to transplant patient follow-up. Whether it is possible to predict whether the dosage of immunosuppressants should be adjusted to prevent antibody production by monitoring CCL22 is unclear, and more clinical data are needed to answer this question.

### Depletion of long-lived plasma cells

Moreover, in AMR patients, traditional immunosuppressive agents do not rapidly eliminate DSAs and cannot block the persistent source of antibodies, namely, long-lived plasma cells. Some drugs targeting plasma cells, such as the proteasome inhibitor-bortezomib, can inhibit the function of long-lived plasma cells and reduce the concentration of serum DSAs [100]. However, bortezomib also has corresponding shortcomings. For example, related studies have shown that compared with that in early AMR, the graft function of advanced patients using bortezomib was not obviously improved [101]. Short-term treatment cannot reduce serum DSA levels. On the one hand, this may be due to the negative feedback mechanism generated by the short-term reduction of plasma cells, which leads to the proliferation of B cells and the production of new ASCs; on the other hand, this may be because the half-life of bortezomib is lower than that of serum DSAs. For the inhibition of plasma cells, DSAs showed false negative results in clinical detection [102,103]. Therefore, the combination of mycophenolic acid, rituximab, and plasma exchange before bortezomib treatment can significantly enhance the therapeutic effect of bortezomib. However, bortezomib can cause side effects such as peripheral neuropathy, gastrointestinal reactions, and cytopenia [104]. When combined with traditional immunosuppressants, we should pay attention to the dose of immunosuppressants.

**Figure 2.**
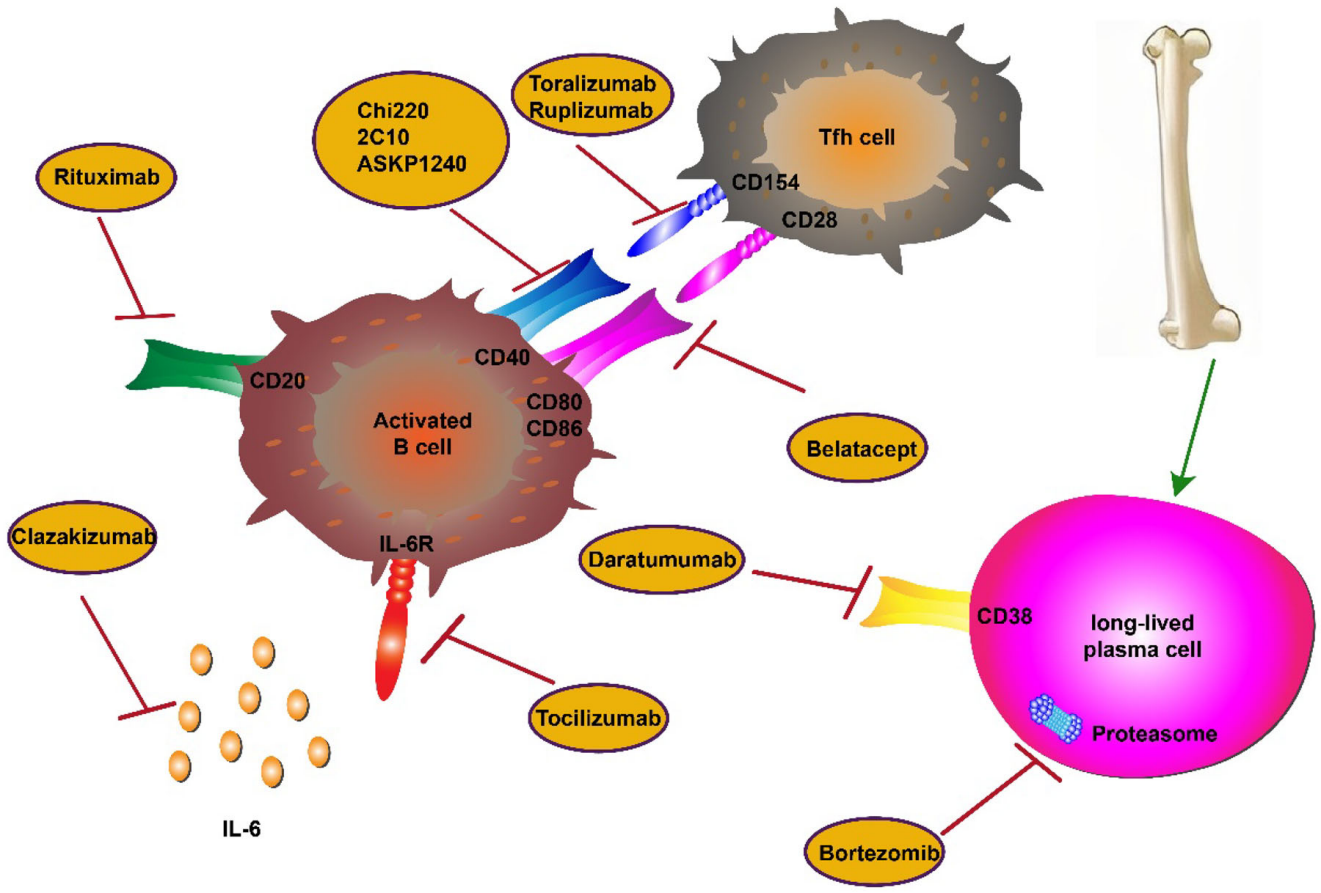
Approaches for the treatment of AMR by targeting T-B-cell interactions and long-lived plasma cells. Rituximab is a monoclonal antibody that acts on the CD20 molecule on the surface of activated B cells. Belatacept inhibits T-cell activation by blocking the CD28-CD80/86 pathway. Toralizumab and ruplizumab are anti-CD154 antibodies, and Chi220, 2C10 and ASKP1240 are anti-CD40 antibodies. These drugs can block the CD40-CD154 pathway and inhibit the production of DSAs. Clazakizumab and tocilizumab are monoclonal antibodies against IL-6 and IL-6R that can block the differentiation of B cells into plasma cells that secrete antibodies. Bortezomib is a proteasome inhibitor that induces apoptosis in long-lived plasma cells. Daratumumab is a CD38 monoclonal antibody that reduces serum antibody concentrations in the short term.

Due to the high expression of CD38 molecules on the surface of long-lived plasma cells, mAbs against CD38 molecules have a certain effect in reducing CD38+ plasma cells. For example, daratumumab, originally used to treat patients with multiple myeloma, is increasingly being studied for its effects on transplant rejection in patients. Konstantin and Jean [105,106] found that daratumumab can reduce serum DSA concentrations and improve graft survival in the short-term treatment of CAMR. However, a common side effect reported in both studies was a rapid rebound in DSAs. Studies have shown that IL-6, an inflammatory factor, binds the IL-6 receptor on activated B cells, which not only promotes the differentiation of B cells into mature plasma cells, but also inhibits the differentiation of Treg cells. It is worth noting that the survival of long-lived plasma cells in the bone marrow is closely related to IL-6 [107,108]. Therefore, blocking the interaction between IL-6 and IL-6R could theoretically inhibit the function of long-lived plasma cells, promote the production of Tregs, and reduce the production of DSAs. Choi et al. [109] showed that in patients with CAMR treated with tocilizumab (a mAb targeting IL-6R), DSA levels were significantly reduced, and the long-term survival rate of transplanted kidneys reached 80%. In addition, an ongoing clinical trial (ClinicalTrials.gov, NCT03380377) explored the safety and tolerance of clazakizumab (an anti-IL-6 mAb) in patients with CABMR and observed changes in the DSA concentration and graft biopsy pathology within 12 months; thus, anti-IL-6 and IL-6R mAbs may become new ways to promote graft stability (Figure 2). In conclusion, the fundamental reason why CAMR is difficult to treat lies in the presence of long-lived plasma cells in the bone marrow. The abovementioned drugs targeting long-lived plasma cells are clinically used in combination with traditional immune preparations to achieve a long-term reduction of the DSA concentration and deplete plasma cells, but more clinical research is required to advance the application of these drugs.

## Conclusions and perspectives

In this review, we describe the pathways through which memory B cells and long-lived plasma cells are produced and some possible strategies for preventing AMR. Currently, for CAMR patients, no single therapeutic target can completely attenuate the impact of transplant organ rejection. Therefore, this article briefly describes some new preventive measures based on memory B cells and long-lived plasma cells, such as the interference of DSA production with chemokines such as CXCL13, CCL22 and PD-1 molecules, which may become intervention targets. In addition, T-cell-B-cell interactions require the participation of many costimulatory molecules (CD28/CD80/CD86 and CD40/CD154), and blocking their interaction may be an effective intervention. Long-lived plasma cells are a long-term source of DSAs, and therapies targeting long-lived plasma cells, such as proteasome inhibitors, CD38 mAbs, and IL6-IL6R blockers, are also effective approaches. There is also heterogeneity among memory B cells, as shown by the differences between IgM^+^ and IgG^+^ memory B cells, with IgG^+^ memory B cells being more responsive to antigens and tending to produce more antibodies when antigens enter the human body. Therefore, quantifying IgM^+^ and IgG^+^ memory B cells is important for following up with transplant rejection patients. Therefore, memory B cells are a uniquely advantageous marker for the future monitoring of AMR. Effective monitoring of memory B cells and long-lived plasma cells before the production of DSAs may be a valid prophylactic measure against AMR. Of course, there are great difficulties associated with meeting this goal and efforts need to be supported with adequate clinical data.
